# Correlates of Ki-67 proliferation index in a cohort of women with suspected breast cancer in Lusaka, Zambia

**DOI:** 10.1371/journal.pgph.0005752

**Published:** 2026-04-06

**Authors:** Joaquim Musamba, David Chisompola, Phinnoty Mwansa, Kingsley Kamvuma, Situmbeko Liweleya, Sepiso K. Masenga

**Affiliations:** 1 HAND Research Group, Mulungushi University, School of Medicine and Health Sciences, Livingstone, Zambia; 2 Division of Integrated Sciences, Livingstone Center for Prevention and Translational Science, Livingstone, Zambia; University of Embu, KENYA

## Abstract

Ki-67 is a key biomarker of tumor proliferation in breast cancer, yet its clinical correlates in this population, where disease is often detected at earlier stages, remain underexplored. This study aimed to identify factors independently associated with high Ki-67 expression among women investigated for suspected breast cancer at Unilabs Laboratory, Lusaka, Zambia. A retrospective cross-sectional analysis was conducted on 208 women suspected of having breast cancer through a laboratory-based screening program in Lusaka, Zambia (2019–2024). Demographic, clinical, and pathology data were extracted from laboratory records. Ki-67 expression, as the outcome variable, was dichotomized as low (<20%) or high (>20%). Variables significant in bivariate analysis (p < 0.05) were included in a multivariable logistic regression model to identify correlates of high Ki-67 expression. The median age was 48 years (IQR: 40–63), and 46.2% (n = 96) exhibited high Ki-67 expression. In bivariate analysis, younger age, invasive ductal carcinoma, right breast involvement, progesterone receptor (PR) positivity threshold ≥10%, non-Quick Score scoring methods, and lack of fluorescence in situ hybridization (FISH) testing were associated with high Ki-67. However, in adjusted multivariable models, only older age (>41 years, aOR: 0.43–0.46, p < 0.05), PR positivity threshold ≥10% (aOR: 6.14–6.38, p < 0.05), and use of non-Quick Score scoring methods (aOR: 10.5–14.9, p ≤ 0.002) remained significantly associated with high Ki-67, while other factors lost statistical significance after controlling for confounders. In this diagnostic cohort from Zambia, nearly half of women with breast cancer exhibited high Ki-67 expression, reinforcing its relevance even in early detection settings. The study identified younger age (≤40 years), higher PR positivity threshold, and alternative scoring methods as independent correlates of high Ki-67, highlighting the importance of standardized biomarker assessment. Future studies should consider a prospective study design incorporating molecular subtyping to enhance the clinical interpretation of Ki-67 in similar populations.

## Introduction

Breast cancer remains the most frequently diagnosed cancer among women worldwide, with an estimated 660,000 deaths [1]. The global burden of breast cancer continues to rise, driven by aging populations, lifestyle changes, and improvements in cancer detection and reporting [2,3]. Robust screening programs are therefore critical for early diagnosis and improved survival outcomes.

The management of breast cancer has been increasingly guided by personalized medicine, relying on biomarker-driven stratification [4]. Established biomarkers, including estrogen receptor (ER), progesterone receptor (PR), and human epidermal growth factor receptor 2 (HER2), are essential for prognostication and for tailoring targeted therapies, thereby enhancing treatment efficacy and minimizing unnecessary toxicity [5–9]. Beyond these, the Ki-67 proliferation index has emerged as an important supplementary biomarker [4,10]. As a nuclear protein expressed during all active phases of the cell cycle, Ki-67 reflects the proportion of proliferating tumor cells, providing a direct measure of tumor aggressiveness. Elevated Ki-67 expression has been consistently linked with more aggressive tumor biology, poorer prognosis, and greater responsiveness to certain chemotherapeutic regimens [11,12].

Despite its clinical relevance, the routine application of Ki-67 faces challenges, particularly regarding standardization and interpretation [4,13]. Most evidence on Ki-67’s prognostic value derives from studies of cohorts with confirmed, often advanced, breast cancer [14–17]. There is limited evidence from sub-Saharan Africa, and particularly in Zambia, on Ki-67 expression patterns in screening populations, where disease stage and biomarker behavior may differ from clinical cohorts. This lack of local, screening-population data hinders the effective integration of Ki-67 into routine prognostic models and treatment decision-making in our setting. Understanding these associations is essential to optimize Ki-67’s utility across the full spectrum of breast disease.

This gap in knowledge presents a challenge for the standardized application of Ki-67 in clinical practice. A more comprehensive understanding of its correlations in a heterogeneous screening population could improve interpretation and contextualization of this biomarker. Therefore, this study aimed to systematically characterize the clinical and pathological factors associated with Ki-67 expression in a cohort of women with clinically suspected breast cancer who underwent diagnostic biopsy at Unilabs, Lusaka, Zambia.

## Methodology

### Design, setting and population

This retrospective cross-sectional study utilized laboratory data from women with suspected breast cancer whose diagnostic samples were processed at Unilabs Laboratory in Lusaka, Zambia, between January 2019 and December 2024. Data were abstracted from electronic medical records between 2 June and 31 July 2025. A total of 210 records were reviewed, of which 208 met the inclusion criteria and were enrolled in the study; two (2) were excluded as they were male participants.

### Inclusion and exclusion criteria

Eligibility criteria were defined to ensure consistency and reliability of the study data. The study included histologically confirmed cases of breast cancer among women with documented expression levels of key biomarkers, namely estrogen receptor (ER), progesterone receptor (PR), human epidermal growth factor receptor 2 (HER2), and the proliferation marker Ki-67. Cases were excluded if biomarker expression data were incomplete or missing. Additionally, patients who had received neoadjuvant chemotherapy or hormone therapy prior to biomarker assessment were excluded to avoid potential alterations in baseline expression levels. Male patients were also excluded from the study.

### Study variables

The variables collected encompassed demographic, clinical, and pathological characteristics. Demographic variables included age and menopausal status. Clinical presentation data captured self-reported or clinically documented information regarding the affected breast side. Pathological variables included final diagnosis (cancer versus non-cancer), histological subtype, and tumor grade. Biomarker-related variables consisted of estrogen receptor (ER), progesterone receptor (PR), and human epidermal growth factor receptor 2 (HER2) status, including staining intensity and the positivity threshold. However, personal identifiers such as names were not retrieved from the electronic registers in order to ensure anonymity of the participants.

Ki-67 expression was assessed immunohistochemically on core biopsy or surgical specimens, and patients were categorized into high or low Ki-67 expression groups based on a predefined institutional cutoff (0: Low Proliferation <20%, 1: High Proliferation >20%) [18]. Additional information regarding the biomarker scoring method (such as Quick Score or alternative systems) and the performance of fluorescence in situ hybridization (FISH) testing for equivocal HER2 results was also recorded.

### Data collection

Clinical and pathology data were retrospectively extracted from electronic medical records from June 2nd to July 31st, 2025, and systematically entered into a standardized electronic report form using the REDCap data management platform. Following extraction, the dataset was exported to Microsoft Excel 2019 for data cleaning and validation prior to statistical analysis.

### Statistical analysis

Data are presented as medians with interquartile ranges (IQR) for continuous variables and frequencies with percentages for categorical variables. The Mann–Whitney U test was used for continuous variables, and the Chi-square or Fisher’s exact test was used for categorical variables to assess bivariate associations with Ki‑67 expression status (dichotomized as low < 20% or high ≥20%). Variables with a p‑value <0.05 in bivariate analysis were entered into multivariable logistic regression models to identify factors independently associated with high Ki‑67 expression. To account for potential confounding and to assess the robustness of the associations, three separate multivariable models were fitted:

- Model 1 adjusted for estrogen receptor (ER) status;
- Model 2 adjusted for progesterone receptor (PR) status;
- Model 3 adjusted for human epidermal growth factor receptor 2 (HER2) status.

Each model included the same core set of covariates: age group (>41 years vs. ≤ 40 years), histology type (invasive ductal carcinoma vs. non‑carcinoma), clinical history (affected side: right vs. left breast), PR positivity threshold (≥10% vs. > 1%), biomarker scoring method (Quick Score vs. other), and fluorescence in situ hybridization (FISH) testing status (yes vs. no). All analyses were performed using Stata version 15, and a p‑value < 0.05 was considered statistically significant.

### Ethical consideration and consent to participate

Ethical clearance for this study was granted by the Mulungushi University School of Medicine and Health Sciences (SOMHS) Research Ethics Committee (Reference No: SMHS-MU2-2025-14) on 6^th^ May 2025, and by the National Health Research Authority (Reference No. NHRA-2208/08/05/2025). The study utilized secondary data extracted from existing medical records; therefore, no personally identifiable information was collected or included in the data collection forms. As the study involved retrospective analysis of anonymized data, the requirement for written or verbal informed consent was waived by the ethics committees.

The study was conducted in accordance with the principles of the Declaration of Helsinki and the Good Clinical Practice (GCP) Guidelines. Reporting adhered to the Strengthening the Reporting of Observational Studies in Epidemiology (STROBE) checklist, as detailed in Supplementary File S1 Checklist. Additionally, the anonymized dataset used for the analysis is provided as supporting information in S1 Data.

## Results

### Patient and tumor characteristics

A total of 208 women were included in this study. The cohort’s demographic, clinical, and pathological characteristics are summarized in Table 1. The median age of the participants was 48 years (IQR: 40, 63). The majority of patients were postmenopausal (62.5%, n = 130) and had a diagnosis of breast cancer (94.2%, n = 196), with invasive ductal carcinoma (IDC) being the predominant histology (94.2%, n = 195). Based on the study’s Ki-67 cutoff, 96 patients (46.2%) were classified as having high Ki-67 expression, while 112 (53.8%) had low expression (Table 1).

**Table 1:** Association between clinical/pathological factors and Ki-67 expression in women.

Characteristic	Median (IQR)/Frequency (%)	Ki-67 Expression		p-value
		High = 96 (46.2%)	Low = 112 (53.8%)	
**Demographics**				
**Age (years)**	48 (40, 63)	46 (38,62.7)	50.6 (42, 63)	0.0767
**Age Group (years)**				
*< 40 years*	57 (27.4)	34 (59.6)	23 (40.4)	**0.016**
*> 41 years*	151 (72.6)	62 (41.1)	89 (58.9)	
**Menopausal Status**				
*Premenopausal*	78 (37.5)	41 (52.6)	37 (47.4)	0.151
*Postmenopausal*	130 (62.5)	55 (42.3)	75 (57.7)	
**Histology Type**				
*IDC*	195 (94.2)	83 (42.6)	112 (57.4)	**<0.0001**
*No Carcinoma*	5 (2.4)	5 (100)	0 (0)	
**Clinical Presentation**				
**Clinical History (Affected Side)**				
*Left Breast*	29 (13.9)	6 (20.7)	23 (79.3)	**0.007**
*Right Breast*	35 (16.8)	15 (42.9)	20 (57.1)	
**Pathology**				
**Tumor Grade**				
*Grade 2*	1 (0.5)	1 (100)	0 (0)	0.170
*Grade 3*	11 (5.3)	3 (27.3)	8 (72.7)	
**Biomarker Status**				
**ER Status**				
*Negative*	84 (40.4)	34 (40.5)	50 (59.5)	0.176
*Positive*	124 (59.6)	62 (50.0)	62 (50.0)	
**PR Status**				
*Negative*	117 (56.3)	52 (44.4)	65 (55.6)	0.575
*Positive*	91 (43.7)	44 (48.4)	47 (51.6)	
**HER2 Status**				
*Negative*	132 (63.5)	62 (47.0)	70 (53.0)	0.756
*Positive*	76 (36.5)	34 (44.7)	42 (55.3)	
**Biomarker Detailed Staining**				
**ER Staining Intensity**				
*Strong*	93 (44.7)	46 (49.5)	47 (50.5)	0.647
*Moderate*	9 (4.3)	5 (55.6)	4 (44.4)	
*Weak*	23 (11.1)	11 (47.8)	12 (52.2)	
**ER Positivity Threshold**				
*> 1%*	19 (9.1)	6 (31.6)	13 (68.4)	0.189
*> 10%*	37 (17.8)	21 (56.8)	16 (43.2)	
**PR Staining Intensity**				
*Strong*	46 (22.1)	26 (56.5)	20 (43.5)	0.430
*Moderate*	8 (3.9)	3 (37.5)	5 (62.5)	
*Weak*	32 (15.4)	13 (40.6)	19 (59.4)	
**PR Positivity Threshold**				
*> 1%*	18 (8.6)	4 (22.2)	14 (77.8)	**0.038**
*> 10%*	17 (8.2)	11 (64.7)	6 (35.3)	
**Scoring Method**				
*Quick Score*	192 (92.3)	82 (42.7)	110 (57.3)	**0.001**
*Other*	16 (7.7)	14 (87.5)	2 (12.5)	
**HER2 Positivity Threshold**				
*> 10%*	21 (10.1)	8 (38.1)	13 (61.9)	0.626
*> 30%*	15 (7.2)	6 (40.0)	9 (60.0)	
**FISH Testing**				
*Yes*	4 (1.9)	1 (25.0)	3 (75.0)	**<0.0001**
*No*	190 (91.4)	82 (43.2)	108 (56.8)	

**Abbreviations:** Median (IQR; Interquartile Range) or Frequency (%; Percentage). P-values for continuous variables (Age) were calculated using the Mann-Whitney U test; p-values for categorical variables were calculated using the Chi-square or Fisher’s exact test, as appropriate. P-values in Bold indicate statistically significance (<0.05).

^a^ER: Estrogen Receptor; PR: Progesterone Receptor; IDC: Invasive ductal carcinoma.

^b^The “No Cancer” group includes benign breast conditions. The “No Carcinoma” and “Other” histology groups include non-malignant diagnoses and non-IDC malignancies, respectively.

### Factors associated with high Ki-67 expression

Univariable and multivariable logistic regression analyses were performed to identify clinicopathological factors associated with high Ki-67 expression. Three separate multivariable models (Models 1, 2 and 3) were constructed, each incorporating a different primary biomarker (ER status, PR status, and HER2 status, respectively) while adjusting for the same core set of variables: age, histology type, tumor laterality, PR positivity threshold, scoring method, and FISH testing status. The results are presented in Table 2. In univariable analysis, which was consistent across all models, several factors were significantly associated with high Ki-67 status. Patient age greater than 41 years was inversely associated with high Ki-67 (odds ratio [OR]: 0.47, 95% confidence interval [CI]: 0.25–0.87, p = 0.017). Positive associations were observed for a progesterone receptor (PR) positivity threshold >10% (OR: 6.41, 95% CI: 1.44–28.5, p = 0.015) and the use of a scoring method other than “Quick Score” (OR: 10.1, 95% CI: 2.26–45.7, p = 0.002) (Table 2). A non-significant trend towards higher Ki-67 was noted for tumors in the right breast (OR: 2.87, 95% CI: 0.93–8.81, p = 0.065). For the histology category “No Carcinoma,” quasi-complete separation of data points occurred, preventing valid odds ratio calculation. The multivariable analyses confirmed the independence of three key predictors, with remarkably consistent effect sizes across all three models (Table 2). Age > 41 years remained a significant protective factor (adjusted OR [aOR] range across models: 0.43 to 0.46; all p < 0.05). Conversely, PR positivity threshold >10% (aOR range: 6.14 to 6.38; all p < 0.05) and use of an alternative scoring method (aOR range: 10.5 to 14.9; all p ≤ 0.002) were strong, independent predictors of high Ki-67 expression. The association for right breast tumors was attenuated and remained non-significant in the multivariable models (aOR range: 2.87 to 3.09; p = 0.050 to 0.065). FISH testing status was not a significant predictor in any model. The issue of quasi-complete separation for the “No Carcinoma” group persisted in the multivariable analyses (Table 2).

**Table 2:** Univariable and multivariable logistic regression analyses of factors associated with high Ki-67 expression.

Variable	OR (95% CI)	p-value	Model 1 (ER) aOR (95% CI)	p-value	Model 2 (PR) aOR (95% CI)	p-value	Model 3 (HER2) aOR (95% CI)	p-value
**Age Group**								
*< 40 years*	Ref	–	Ref	–	Ref	–	Ref	–
*> 41 years*	0.47 (0.25, 0.87)	**0.017**	0.46 (0.24, 0.86)	**0.016**	0.45 (0.24, 0.85)	**0.014**	0.43 (0.23, 0.83)	**0.012**
**Histology**								
IDC	Ref	–	Ref	–	Ref	–	Ref	–
No Carcinoma	†	–	†	–	†	–	†	–
**Clinical History (Side affected)**								
*Left Breast*	Ref	–	Ref	–	Ref	–	Ref	–
*Right Breast*	2.87 (0.93, 8.81)	0.065	3.09 (0.99, 9.57)	0.050	2.89 (0.94, 8.90)	0.063	2.87 (0.93, 8.81)	0.065
**PR Threshold**								
*> 1%*	Ref	–	Ref	–	Ref	–	Ref	–
*> 10%*	6.41 (1.44, 28.5)	**0.015**	6.14 (1.37, 27.3)	**0.017**	6.16 (1.37, 27.6)	**0.017**	6.38 (1.43, 28.5)	**0.015**
**Scoring Method**								
*Quick Score*	Ref	–	Ref	–	Ref	–	Ref	–
*Other*	10.1 (2.26, 45.7)	0.002	14.9 (3.18–70.2)	0.001	12.2 (2.65–56.1)	0.001	10.5 (2.32–48.2)	0.002
**FISH Testing**								
*Yes*	Ref	–	Ref	–	Ref	–	Ref	–
*No*	2.27 (0.23, 22.2)	0.479	3.10 (0.31, 30.6)	0.333	2.23 (0.23–23.0)	0.470	2.41 (0.24–23.8)	0.451

Abbreviations: OR, odds ratio; aOR, adjusted odds ratio; CI, confidence interval; ER, estrogen receptor; HER2, human epidermal growth factor receptor 2; IDC, invasive ductal carcinoma; PR, progesterone receptor. Note: All multivariable models were adjusted for all variables listed in the table, with the primary biomarker (ER, PR, or HER2 status) integrated as appropriate for each model. † Odds ratio not calculable due to quasi-complete separation. It should be noted that certain subgroups (e.g., “No Carcinoma,” FISH testing) contained small numbers of observations, leading to wide confidence intervals in the regression models. These estimates should be interpreted with caution.

## Discussion

This study aimed to identify factors associated with a high Ki‑67 proliferation index among women investigated for suspected breast cancer in Lusaka, Zambia. The analysis revealed several significant associations in univariable models, and three factors remained independently associated with high Ki‑67 in adjusted multivariable analyses: age > 41 years (indicating a protective effect), a progesterone receptor (PR) positivity threshold of ≥10%, and the use of a biomarker scoring method other than the Quick Score. These findings provide new insights into the clinicopathological correlates of Ki‑67 expression in a diagnostic cohort from sub‑Saharan Africa and underscore both the relevance and the complexity of interpreting this biomarker in clinical practice.

Our study reveals a 46.2% prevalence of high Ki-67 expression in this diagnostic cohort of women with breast cancer. This proportion is lower than the 56.5% and 50.5% reported in the monarchE Phase 3 clinical trial [19,20]. This variation may reflect differences in tumor biology, population, or the Ki‑67 scoring thresholds applied. In contrast, it is higher than the 33.8% reported in a study from Pakistan [21], suggesting potential geographic, genetic, or environmental influences on tumor proliferative activity. Overall, nearly half of the screened women in our setting exhibited high‑proliferation tumors, reinforcing the importance of Ki‑67 as a prognostic biomarker even in early‑detection contexts.

Age emerged as an independent predictor of high Ki‑67 expression in our cohort, with women over 41 years exhibiting a lower likelihood of high proliferative activity. This association may reflect several age‑related biological and clinical factors. Firstly, younger women are more likely to develop aggressive, high‑grade tumors with elevated proliferative indices, a pattern consistent with global reports of more adverse tumor biology in premenopausal populations [22]. Secondly, in a diagnostic cohort such as ours, younger women may present with symptomatic or palpable disease, potentially enriching for faster‑growing, clinically evident tumors. Although we adjusted for menopausal status, the complex interplay between age, hormonal milieu, and tumor phenotype may not be fully captured. Furthermore, the age distribution in our cohort, with a median of 48 years and limited representation of extremes, may affect the generalizability of these findings. Therefore, future studies with broader age representation and detailed molecular subtyping are warranted to clarify the biological and clinical implications of age‑related differences in Ki‑67 expression.

The strong independent association between a PR positivity threshold of ≥10% and high Ki‑67 (aOR range: 6.14–6.38, p < 0.05) is noteworthy, as PR expression is typically linked to less aggressive, hormone‑responsive disease [23]. This counter‑intuitive finding may reflect unique tumor biology in our population or indicate that high PR expression, when coupled with high proliferation, identifies a distinct subgroup with altered hormonal signaling. While PR expression is typically linked with hormone‑responsive, less aggressive disease, our finding may reflect unique tumor biology within this Zambian cohort, differential antibody staining protocols, or threshold‑specific interactions between hormonal signaling and proliferation pathways. It should be noted, however, that the absence of molecular subtyping in our cohort limits the biological contextualization of this finding. Further studies incorporating molecular subtyping and standardized receptor scoring are needed to clarify whether this represents a biologically distinct phenotype or a methodological artifact.

The study findings reveal the association between a scoring method “other” and high Ki-67 expression (aOR range: 10.5–14.9, p ≤ 0.002) likely reflects methodological variability rather than true tumor biology. This is particularly relevant given the small number of cases assessed with alternative methods (n = 16). This finding highlights a critical quality‑assurance challenge in biomarker assessment. Inconsistencies in scoring practices, whether due to differing interpretation guidelines, laboratory protocols, or pathologist experience, can profoundly influence Ki‑67 categorization. Such variability reduces comparability across clinical and research settings. Our finding underscores the important need for standardized, validated scoring systems to ensure reliable and reproducible Ki‑67 reporting, especially in resource‑limited diagnostic environments.

Clinically, high Ki‑67 expression is a well‑established marker of tumor aggressiveness, correlating with poorer prognosis, higher histological grade, and increased risk of recurrence [24–27]. Our results affirm that Ki‑67 remains a relevant biomarker in a Zambian screening population. However, the strong dependence on scoring methodology observed here calls for caution in its clinical application. Without local standardization, Ki‑67 results may be misleading and could potentially affect treatment decisions, particularly in resource‑limited settings where access to advanced molecular subtyping is limited.

### Strengths and limitations

This study has several strengths, including a well‑characterized cohort, rigorous multivariable modeling adjusted for key biomarkers, and transparent reporting of both univariable and multivariable associations. However, important limitations must be acknowledged. The cross‑sectional design precludes causal inference. Quasi‑complete separation for the “No Carcinoma” category limited the inclusion of this group in regression models. Several subgroup analyses, particularly those involving rare categories such as non‑carcinoma diagnoses, alternative scoring methods, and FISH testing, were based on small numbers, resulting in wide confidence intervals; these findings should therefore be interpreted with caution. The absence of molecular subtyping data restricts our ability to contextualize Ki‑67 within established intrinsic subtypes. Additionally, the retrospective reliance on laboratory records may have introduced variability in biomarker assessment and documentation. Future prospective studies, incorporating standardized scoring protocols and molecular classification, are needed to validate these findings and better define the role of Ki‑67 in similar populations.

## Conclusion

In this Zambian diagnostic cohort, nearly half of the women exhibited high Ki‑67 expression. Younger age, a higher PR positivity threshold, and the use of non‑Quick Score scoring methods were associated with high Ki‑67 expression. These findings underscore both the clinical relevance of Ki‑67 and the critical importance of standardized biomarker assessment in ensuring its reliable interpretation. Efforts to harmonize scoring practices and integrate molecular subtyping in future studies will enhance the utility of Ki‑67 in prognostic stratification and treatment planning in sub‑Saharan African settings.

## Supporting information

S1 Checklist STROBE checklist. This checklist is adapted from the STROBE Statement (von Elm et al., 2007, PLoS Medicine; https://doi.org/10.1371/journal.pmed.0040296), available under a Creative Commons Attribution 4.0 International License (CC BY 4.0).
(DOCX)

S1 Data Raw data used to generate results for this manuscript. (XLSX)
